# Interstitial Ion Regulation in Cognitive Fatigability and Delayed Recovery: A Hypothesis-Driven Critical Review

**DOI:** 10.3390/ijms27198508

**Published:** 2026-09-24

**Authors:** Hiromu Monai

**Affiliations:** Institute of Human Life Sciences, Ochanomizu University, Bunkyo-ku 112-8610, Tokyo, Japan; monai.hiromu@ocha.ac.jp

**Keywords:** cognitive fatigability, brain fatigue, extracellular potassium, interstitial ionic state, astrocyte, extracellular ion homeostasis, non-synaptic signaling, delayed recovery

## Abstract

Cognitive fatigability is the time-dependent destabilization of cognitive performance under sustained demand and is distinct from subjective fatigue. Neurophysiological effects may persist after demand. To link performance instability during demand with delayed recovery, this non-systematic, hypothesis-driven Review focuses on the extracellular milieu, which reflects and shapes neural activity. This Review operationally defines the interstitial ionic state as extracellular K^+^, Ca^2+^, Mg^2+^, pH, and extracellular space volume. Neurons, glia, and the vasculature generate this state; astrocytes couple neuromodulatory input to K^+^ and glutamate transport, metabolism, and water movement. Astrocyte involvement should be assessed through transport and extracellular variables rather than Ca^2+^ alone. This Review proposes that sustained cognitive demand constrains maintenance of this milieu during information processing and reconfiguration afterward. Delayed recovery may reflect incomplete reconfiguration of the milieu or incomplete recovery of neuronal and astrocytic responsiveness. Within the literature examined, no direct test of this integrated hypothesis under ordinary sustained cognitive demand was identified. The hypothesis predicts that recovery trajectories will outperform single post-demand measurements in forecasting renewed-demand performance and that recovery-phase manipulation of ion regulation or cellular responsiveness will alter fatigability. Brain fatigue may partly reflect reduced capacity to maintain and flexibly reconfigure the extracellular milieu across brain states.

## 1. Introduction

Cognitive fatigability is distinguished from subjective fatigue as a time-dependent deterioration in objectively measured cognitive performance under a defined cognitive demand [1]. During prolonged cognitive tasks, frontal activity interpreted as compensatory can increase before accuracy or reaction time clearly deteriorates, with performance declining after this activity subsides [2]. Following a working-memory task, endogenous brain dynamics required several minutes to approach their pre-task temporal organization, and recovery was slower after the higher-load condition [3]. Daylong cognitive work has also been associated with glutamate-related magnetic resonance spectroscopy (MRS) signals in the lateral prefrontal cortex and altered allocation of effort [4]. Together, these studies indicate that sustained cognitive demand can be accompanied by compensatory activity before overt performance decline, altered effort allocation, and neurophysiological effects that persist after task completion.

A unified neurophysiological account should explain how information processing is initially sustained, why its stability is progressively lost, and how the physiological consequences of demand affect the transition toward rest, sleep, or renewed cognitive demand. Cognitive fatigability during demand and delayed recovery afterward are distinct phenomena, but they should be considered as connected phases of a continuous process.

### 1.1. Literature Search and Scope

This hypothesis-driven critical Review was not designed as a systematic review or meta-analysis. Searches focused on PubMed, supplemented by publisher websites, Crossref/CrossMark records, and backward citation tracking, and covered English-language literature through 6 September 2026. Primary studies were prioritized for cellular mechanisms, whereas reviews were used mainly for conceptual orientation. Sleep–wake and movement studies were treated as evidence for physiological principles, brain-slice and genetic studies as evidence for cellular mechanisms, and ischemia and cortical spreading depolarization as boundary cases rather than direct models of ordinary cognitive demand. Human studies were retained primarily to define cognitive fatigability, compensation, and post-demand recovery. Negative-claim searches combined terms related to cognitive fatigue or fatigability, mental fatigue, sustained cognitive demand, extracellular or interstitial potassium and ions, astrocytes, extracellular space, and recovery. Statements that no direct report was identified refer only to the literature examined and do not establish complete absence. Disease-specific fatigue, formal risk-of-bias assessment, and quantitative synthesis were outside the scope of this Review. During manuscript preparation, ChatGPT (OpenAI, GPT-5.6 Pro; accessed July 2026) was used for structural editing, English-language rewriting, reference cross-checking, and the preparation of conceptual figures. It was not used as a primary source of scientific evidence. All outputs were reviewed and edited by the author, who takes full responsibility for the manuscript.

### 1.2. Rationale and Working Hypothesis

Inflammatory and metabolic findings illustrate both the relevance and the limitations of current candidate mechanisms. In healthy participants, experimental inflammation increased subjective fatigue and sleepiness [5], whereas a separate study found reduced selection of high-effort options [6]. These results show that inflammation can alter subjective symptoms and effort allocation, but they do not establish neuroinflammation as the mechanism of cognitive fatigability under ordinary cognitive demand. The glutamate-related MRS finding is likewise not mechanistically specific: the method used by Wiehler and colleagues did not directly resolve cell type or intra- versus extracellular compartments, so the observed signal cannot be assigned uniquely to extracellular glutamate accumulation, altered metabolism, or altered transport [4].

Taken individually, these findings do not yet explain how cognitive performance can be preserved early in a demand, become unstable later, and recover only gradually after the demand ends. A more integrative account requires a physiological level that both carries the effects of ongoing activity and alters the conditions for subsequent activity.

The extracellular milieu is a candidate for such a level. Action potentials and synaptic transmission alter extracellular K^+^, Ca^2+^, Mg^2+^, pH, neurotransmitter concentrations, and extracellular space volume; these changes, in turn, influence neuronal membrane potential, spiking, and synaptic transmission. Sleep–wake studies provide a concrete example: extracellular ions and extracellular space change coordinately with brain state, and locally imposing a wake-like ionic composition on the sleeping cortex shifts local cortical activity toward a wake-like pattern [7]. These observations make the extracellular milieu a plausible level at which sustained information processing and subsequent recovery could be linked.

In this Review, the term interstitial ionic state refers operationally to the combination of extracellular K^+^, Ca^2+^, Mg^2+^, extracellular pH, and extracellular space volume. This is an operational construct, not an established physiological classification. These variables were selected because they change coordinately across sleep–wake states, experimentally altering their composition changes local neural activity, and they directly shape excitability or the concentration change produced by ionic flux [7]. Although extracellular space volume is not an ion, it determines the concentration change produced by a given ionic flux and is therefore included in the definition. Other extracellular constituents, including Na^+^, Cl^−^, glutamate, other neurotransmitters, adenosine, lactate, glucose, oxygen, and inflammatory mediators, remain part of the broader extracellular milieu but are outside this selected core set. Temperature and osmolarity are additional physical determinants of that milieu. Intracellular Na^+^ and Cl^−^ are cellular-state variables rather than extracellular coordinates. The interstitial ionic state is shaped by ion and solute transport, metabolism, and water movement across neurons, glia, and the vasculature.

Within this multicellular system, astrocytes warrant particular attention because they receive neuromodulatory signals and link K^+^ and glutamate uptake to energy metabolism and water movement. Astrocytes do not control the extracellular milieu alone, but they may contribute both to its maintenance during sustained cognitive demand and to its reconfiguration after the demand ends.

On this basis, this Review proposes that sustained cognitive demand progressively constrains the capacity to maintain an extracellular milieu that supports information processing during demand. A related but distinct proposition concerns delayed reconfiguration of that milieu afterward for rest, sleep, or renewed demand. Extracellular K^+^ is one major variable because it directly influences neuronal and astrocytic membrane potential. Physiological K^+^ elevations, however, also support normal arousal and information processing. This account therefore does not equate cognitive fatigability with simple K^+^ accumulation, uniform astrocyte dysfunction, global energy depletion, or neuroinflammation. The direct targets of explanation are cognitive fatigability during sustained cognitive demand and delayed neurophysiological recovery after the demand and during renewed demand. Here, delayed neurophysiological recovery refers to persistent post-demand effects on brain dynamics, the extracellular milieu, or cellular responsiveness. Brain fatigue is used only as a broader umbrella term, not as a synonym for cognitive fatigability.

## 2. Interstitial Ion Signaling as a Physiological Basis of Brain State

To contribute to an account of cognitive fatigability and delayed neurophysiological recovery, the interstitial ionic state must be more than a by-product of neural activity. Three propositions are relevant: the variables included in this state should change reproducibly with brain state; experimentally altering their composition should influence neural activity; and at least some components should be shaped by neuromodulatory and cellular transport mechanisms. Evidence for these propositions would position the interstitial ionic state as a physiological condition of information processing, although not yet as a demonstrated mechanism of cognitive fatigability.

### 2.1. The Interstitial Ionic State Varies with Brain State

Sleep–wake studies provide direct evidence that the variables included in the interstitial ionic state change with brain state. In mice, the transition from sleep to wakefulness is accompanied by increases in extracellular K^+^ and pH and decreases in extracellular Ca^2+^, Mg^2+^, and extracellular space volume. Transitions into natural sleep or anesthesia are accompanied by changes in the opposite direction [7]. Sleep and wakefulness therefore differ not by a single ion concentration, but by coordinated extracellular ionic and volumetric conditions.

At least one component of this state, extracellular K^+^, also changes within wakefulness. When a stationary mouse begins walking or running, extracellular K^+^ increases by approximately 0.6–1.0 mM across sensory and motor cortices, and the rise begins before movement onset [8]. This timing argues against an explanation based solely on cumulative K^+^ efflux generated after movement begins.

Together, these studies establish coordinated multivariable differences across sleep and wakefulness and state-linked K^+^ changes within wakefulness. Within the literature examined for this Review, no direct measurements of comparable multivariable changes during ordinary sustained cognitive demand were identified. The movement-associated K^+^ rise should therefore be treated as a physiological feature of the awake brain rather than as evidence of abnormal accumulation. The relevant question is whether a state-appropriate extracellular milieu can be formed, maintained, and subsequently reconfigured.

### 2.2. Extracellular Ionic Composition Influences Neural Activity

Covariation with brain state alone could be epiphenomenal. A stronger test is whether experimentally altering extracellular ionic composition changes neural activity.

Locally applying a wake-like combination of K^+^, Ca^2+^, Mg^2+^, and extracellular pH to the cortex of sleeping mice shifted local electrical activity from a sleep-like toward a wake-like pattern [7]. The manipulation did not produce complete whole-animal awakening, but it showed that extracellular ionic composition can alter the local mode of neural activity.

Experimentally reproducing the magnitude of extracellular K^+^ elevation observed during the transition from rest to locomotion depolarized neurons and enhanced their responses to the same input. In awake mice, comparable K^+^ elevations enhanced visually evoked responses in visual cortex and increased neural activity and motor output in motor cortex [8].

The effects of local extracellular K^+^ extend beyond nearby neuronal membrane potential. In the striatum, local K^+^ manipulation altered noradrenaline, dopamine, and serotonin dynamics. In cortex, it altered noradrenaline dynamics, network state, and behavior [9]. Extracellular K^+^ may therefore influence wider brain activity through reciprocal interactions with neuromodulatory systems.

These studies examined sleep–wake transitions, movement, and sensory processing rather than cognitive fatigability during sustained cognitive demand or post-demand neurophysiological recovery. They nevertheless support the principle that extracellular ionic changes within a physiological range can both accompany brain states and alter subsequent neural activity.

### 2.3. Neuromodulatory Systems and the Interstitial Ionic State Interact Bidirectionally

The interstitial ionic state is not formed solely by the accumulation of ions released during neuronal activity. In cortical slices, neuromodulators associated with wakefulness changed extracellular K^+^ even when action potentials were suppressed with tetrodotoxin. In vivo, the wake-associated K^+^ increase could not be accounted for by AMPA receptor-mediated excitatory transmission alone [7]. Together, these findings support a contribution of neuromodulatory regulation to the formation of state-dependent extracellular K^+^ conditions. The tetrodotoxin result does not exclude action-potential-independent transmitter release or identify the cellular source of the K^+^ change.

Noradrenaline also changes astrocytic Ca^2+^ responses to local circuit activity. In the visual cortex of awake mice, comparable local input elicited larger astrocytic Ca^2+^ responses when it was accompanied by noradrenaline release [10].

In the somatosensory system of male mice, serotonin, noradrenaline, and acetylcholine altered post-stimulus extracellular K^+^ clearance in different directions. Kir4.1, Na^+^/K^+^-ATPase, and glycolysis contributed to these effects to different degrees [11]. Thus, even when the regulated variable is the same, the cellular mechanisms and temporal profile depend on the neuromodulatory context.

Noradrenergic signaling also participates in the temporal organization of cortical state. Noradrenergic input promotes cortical desynchronization during arousal, whereas signaling through astrocytic α_1_A-adrenergic receptors contributes to subsequent resynchronization [12]. Astrocytes may therefore participate in the temporal reorganization of cortical activity rather than simply amplifying wakefulness.

Conversely, local changes in extracellular K^+^ influence neuromodulator release [9]. Together, these findings support a bidirectional model in which neuromodulatory input shapes cellular ion regulation and the resulting extracellular conditions feed back on neuronal activity and neuromodulator release. The complete reciprocal interaction has not been demonstrated within a single experiment; it is a mechanistic synthesis of effects established in different preparations.

### 2.4. Astrocytes Link Neuromodulatory Input to Regulation of the Extracellular Milieu

For neuromodulatory input to alter the interstitial ionic state, cells must translate that input into changes in ion and solute transport, metabolism, and water movement.

Astrocytes express neuromodulator receptors and intracellular signaling pathways, as well as K^+^ channels, Na^+^/K^+^-ATPase, and glutamate transporters [10,11,12,13]. They also participate in energy metabolism [14,15] and ion-coupled changes in water movement and cell volume [16,17,18]. Across distinct experimental systems, these properties position astrocytes to couple neuromodulatory input to K^+^ and glutamate transport, metabolic support, and water movement.

Astrocytes are not the sole regulators of the interstitial ionic state. Neuronal Na^+^/K^+^-ATPase restores ionic gradients, and reverse-mode KCC2 can contribute to the clearance of postsynaptically released K^+^ near excitatory synapses [19]. In white matter, oligodendrocyte Kir4.1 limits K^+^ accumulation and seizure susceptibility [20]. In the optic nerve, extracellular K^+^ regulates metabolic coupling between oligodendrocytes and axons [21]. The vasculature and the architecture of extracellular space also shape ionic distribution.

Astrocytes are therefore emphasized not as sole managers of extracellular K^+^, but as cells positioned to link neuromodulatory input with K^+^ and glutamate transport, energy metabolism, and water movement. The direction and magnitude of these effects can vary with the neuromodulator, brain region, and duration of activity. Astrocyte involvement should therefore be assessed through the relevant transport and extracellular variables rather than through Ca^2+^ activity alone.

### 2.5. Homeostatic Maintenance and State Transition Are Two Aspects of the Same Regulatory Process

The term interstitial ion signaling is used here operationally and does not denote a classical chemical signal in which a defined ligand binds a dedicated receptor. Instead, it refers to the influence of a coordinated, spatially distributed physicochemical milieu on membrane potential, ion channels, transporters, and transmitter release across populations of neurons and glia.

The evidence reviewed above supports three features of this framework: coordinated variation with brain state, causal effects of experimentally altered local extracellular composition on neural activity, and regulation of constituent variables by neuromodulatory and cellular mechanisms. These features do not establish a mechanism of cognitive fatigability. Rather, they provide an operational framework for distinguishing physiological state transitions from disordered loss of ionic homeostasis.

Homeostatic maintenance and state transition are not opposing functions. Forming a state-appropriate extracellular milieu changes the conditions under which neurons and glia respond; constraining those changes in magnitude and duration and reconfiguring the milieu as brain state changes are parts of the same regulatory process. A normal extracellular milieu is therefore not a single fixed point, but a state-dependent configuration.

Within this multivariable milieu, extracellular K^+^ acts directly on neuronal and astrocytic membrane potential. Physiological K^+^ changes can support information processing in awake states, but their consequences may depend on activity duration and recent history. The mechanistic problem is therefore how the effects of physiological K^+^ changes evolve over time, rather than whether K^+^ simply rises.

## 3. Extracellular K^+^ Dynamics as a Candidate Proximal Mechanism of Cognitive Fatigability

Among the components of the interstitial ionic state, extracellular K^+^ provides a relatively proximal link to cognitive fatigability because it acts directly on neuronal and astrocytic membrane potential through the K^+^ equilibrium potential, with consequences for distance to firing threshold, voltage-gated channel availability, action-potential propagation, synaptic transmission, and glutamate transport. It is therefore a major extracellular variable through which changes in the extracellular milieu could influence the stability of information processing.

Extracellular K^+^ cannot, however, be treated as a fatigue substance whose effects increase monotonically with concentration. Physiological K^+^ elevation is a normal feature of arousal and information processing. The relevant issue is whether K^+^ can be regulated within a state-appropriate extracellular milieu during sustained cognitive demand and whether recent activity changes the effect of a given K^+^ concentration.

### 3.1. Physiological Elevation of Extracellular K^+^ Supports Information Processing

K^+^ leaves neurons during action-potential repolarization. During excitatory synaptic transmission, K^+^ efflux through postsynaptic NMDA receptors also contributes to local perisynaptic elevations in extracellular K^+^ [22]. Ongoing neural activity is therefore accompanied by a continuing requirement to regulate K^+^ entering the extracellular compartment.

As described in Section 2.1, the locomotion-associated cortical K^+^ rise began before movement onset. Experimentally reproducing a comparable increase depolarized neurons and enhanced visual- and motor-related responses [8]. These findings show that modest physiological K^+^ elevations can support state-dependent neural responsiveness rather than serving only as disturbances to be corrected.

Physiological K^+^ elevation should therefore be distinguished from pathological or poorly controlled accumulation. The question for cognitive fatigability is not whether extracellular K^+^ rises, but whether its magnitude and time course remain compatible with stable information processing as demand continues.

### 3.2. The Effect of K^+^ Depends on Recent Neural Activity

The effect of extracellular K^+^ on neuronal activity is non-monotonic. A modest increase can bring membrane potential closer to threshold and enlarge the response to a given input. Sufficiently large or sustained depolarization, however, can destabilize action-potential amplitude, propagation, and timing.

In hippocampal CA1, raising extracellular K^+^ to 8–10 mM reduced action-potential amplitude during repetitive stimulation and increased conduction failure in a subset of axons [23]. These concentrations are substantially higher than the physiological changes expected during ordinary wakefulness or cognitive demand. This study therefore provides a boundary case rather than a direct model of cognitive fatigability: sufficiently large K^+^-dependent depolarization can shift from facilitating activity to destabilizing it.

Separately, repetitive firing reduces Na^+^ channel availability even in the absence of experimentally elevated extracellular K^+^. In hippocampal CA1 pyramidal neurons, repeated action potentials progressively inactivated dendritic Na^+^ channels and attenuated subsequent back-propagation. Recovery from inactivation was slower than its development and was accelerated by membrane hyperpolarization [24,25]. These findings do not show that physiological K^+^ elevation directly causes Na^+^ channel inactivation. They raise the possibility that K^+^-dependent depolarization could prolong reduced channel availability after repetitive firing.

Repeated firing also increases intracellular Na^+^, activates Na^+^/K^+^-ATPase, and generates an afterhyperpolarization lasting from seconds to tens of seconds [26]. This process restores Na^+^ and K^+^ gradients while transiently reducing neuronal excitability in proportion to recent firing.

If these fast processes recur during sustained cognitive demand, neuronal responses could reflect the changing overlap of K^+^-dependent depolarization, firing-history-dependent Na^+^ channel availability, and pump-mediated hyperpolarization rather than extracellular K^+^ alone. The time-scale mismatch is critical: local K^+^ changes and Na^+^ channel inactivation arise over milliseconds to seconds, and pump-mediated afterhyperpolarization lasts seconds to tens of seconds, whereas cognitive fatigability develops over tens of minutes to hours. Within the literature examined for this Review, no direct demonstration was identified of how repeated short-lived processes are integrated into prolonged instability during sustained cognitive demand. Bridging these timescales may therefore require one or more history-dependent intermediate processes.

### 3.3. Astrocytic K^+^ Regulation Becomes Especially Relevant During Repetitive Activity

Extracellular K^+^ is not regulated by astrocytes alone; neuronal Na^+^/K^+^-ATPase, KCC2, and other mechanisms also contribute. Astrocytes are nevertheless relevant because Kir4.1 contributes to their high K^+^ permeability and hyperpolarized membrane potential, properties that support K^+^ regulation during repeated activity.

Conditional loss of astrocytic Kir4.1 markedly depolarized astrocytes and reduced both K^+^ and glutamate uptake. Basal membrane properties and basal synaptic transmission in CA1 pyramidal neurons were comparatively preserved, whereas short-term synaptic responses after high-frequency stimulation were substantially altered [27]. This contrast illustrates how impaired K^+^ regulation can remain inconspicuous under basal conditions yet become functionally evident during repetitive activity. However, the study used a developmental conditional knockout with severe neurological phenotypes and cannot be extrapolated directly to ordinary cognitive demand [27].

In hippocampal CA1 slices, a sustained astrocytic current evoked by synaptic stimulation was dominated by Kir4.1-mediated K^+^ current and depended on preceding stimulation. Altering astroglial K^+^ clearance changed subsequent synaptically evoked responses [28]. These findings show that local K^+^ regulation can carry the influence of recent neural activity into subsequent synaptic processing.

Acute-slice experiments using local manipulation of astrocytic Kir4.1 expression further showed that Kir4.1 levels controlled the territorial kinetics of K^+^ and glutamate uptake. At synapses within territories of astrocytes with increased Kir4.1 expression, the activity-dependent enhancement of presynaptic Ca^2+^ entry and transmitter release associated with elevated K^+^ was attenuated [29].

Together, these studies suggest that astrocytic K^+^ regulation can be spatially heterogeneous and more consequential during repetitive activity than under basal conditions. They do not establish that such heterogeneity produces cognitive fatigability in awake animals. That behavioral connection remains a working hypothesis.

### 3.4. K^+^ and Glutamate Reciprocally Shape the Local Extracellular Milieu

The dependence of glutamate uptake on Kir4.1-supported astrocyte membrane potential and the transmembrane Na^+^ gradient is considered in Section 4.2.

During repetitive excitatory transmission, K^+^ efflux through postsynaptic NMDA receptors can elevate local extracellular K^+^. The resulting K^+^ elevation can depolarize presynaptic terminals, promote Ca^2+^ entry and additional glutamate release, and depolarize nearby astrocytic processes, thereby reducing the driving force for glutamate uptake [22].

Glutamatergic signaling can therefore contribute to local K^+^ elevation, while the resulting K^+^ change can influence both glutamate release and clearance. This reciprocal coupling creates the potential for local amplification, although K^+^ and glutamate transport normally constrain its spatial and temporal extent. A model of cognitive fatigability does not require pathological accumulation of either K^+^ or glutamate.

Interpreting extracellular K^+^ as a proximal variable therefore requires identifying the extracellular and cellular conditions that shape the effect of a given K^+^ change.

### 3.5. Linking Extracellular K^+^ Dynamics to Cognitive Fatigability

Existing studies support several component mechanisms under distinct experimental conditions. Physiological K^+^ elevation can moderately depolarize neurons and enhance their responses [8]. High-K^+^ boundary conditions can destabilize action-potential conduction [23]. Repetitive firing can reduce Na^+^ channel availability [24,25] and activate Na^+^/K^+^-ATPase-dependent afterhyperpolarization [26]. Kir4.1-dependent K^+^ regulation can influence glutamate uptake and synaptic responses during repetitive stimulation [27,28,29]. These findings were obtained across awake movement and sensory paradigms, brain slices, and genetic or local expression manipulations. Within the literature examined for this Review, no study was identified that measured these processes together during ordinary sustained cognitive demand and directly related them to cognitive performance.

The proposed connection is therefore a working hypothesis. Repeated physiological K^+^ changes during sustained cognitive demand may interact with firing-history-dependent Na^+^ channel availability, pump activity, and astrocytic K^+^ and glutamate regulation. Across many trials, the changing relation among these processes could make the magnitude and timing of neuronal responses less stable without requiring pathological accumulation of K^+^ or glutamate.

This hypothesis does not posit failure of K^+^ regulation at the onset of demand. Physiological K^+^ elevation and neuronal and glial transport may initially support information processing. The unresolved step is whether sustaining comparable processing as demand continues progressively changes the requirements for K^+^ and glutamate transport, restoration of ionic gradients, and the energy supply supporting those processes.

The effect of extracellular K^+^ cannot therefore be interpreted from K^+^ concentration alone. It depends on the extracellular milieu in which the K^+^ change occurs and on the physiological state of the neurons and glia that respond to and regulate that milieu.

## 4. The Extracellular Milieu That Shapes the Effects of K^+^

The contextual determinants of a K^+^ change include extracellular Ca^2+^, Mg^2+^, and pH; extracellular space volume; and astrocyte membrane potential, the transmembrane Na^+^ gradient, and energy supply. Together, these variables shape transmitter release, receptor function, the concentration change produced by a given ionic flux, and the rate of K^+^ and glutamate transport. K^+^ should therefore be interpreted as a major extracellular variable only within the multivariable milieu in which it acts and the cellular state that regulates that milieu.

### 4.1. Extracellular Ca^2+^, Mg^2+^, and pH Shape the Effects of K^+^

Sleep–wake transitions illustrate the multivariable nature of the extracellular milieu. Wakefulness is accompanied not only by increased extracellular K^+^, but also by increased pH, decreased extracellular Ca^2+^ and Mg^2+^, and reduced extracellular space volume; sleep and anesthesia are accompanied by changes in the opposite direction [7]. The wake-associated milieu is therefore not simply a state of elevated K^+^.

Extracellular Ca^2+^ is a major determinant of presynaptic release probability. In hippocampal CA1, repetitive synaptic stimulation transiently depleted extracellular Ca^2+^ and reduced subsequent glutamate release [30]. In distinct cultured preparations, patch-clamp recordings from embryonic mouse mesencephalic or striatal neurons [31] and voltage-clamp recordings from embryonic mouse spinal neurons [32] established that extracellular Mg^2+^ blocks NMDA receptor-linked channels in a membrane-potential-dependent manner.

Extracellular pH also modifies excitatory transmission, but the effects depend on the experimental manipulation. In acute CA1 slices from male Wistar rats, inhibition of extracellular carbonic anhydrase with benzolamide enhanced activity-dependent alkaline transients and potentiated NMDA receptor-mediated transmission, whereas increasing extracellular H^+^ buffering with HEPES attenuated pharmacologically isolated NMDA responses [33]. In a separate study using acute CA1 slices from 5–6-week-old male Sprague–Dawley rats, lowering extracellular pH from 7.4 to 6.7 reduced field excitatory postsynaptic potentials, population spikes, presynaptic fiber volleys, responses to exogenously applied NMDA, and sustained repetitive firing. Postsynaptic responses to exogenously applied AMPA and the induction and maintenance of long-term potentiation were not significantly altered [34].

These studies were conducted in different preparations and do not establish extracellular Ca^2+^, Mg^2+^, or pH as independent causes of cognitive fatigability. Rather, they show that these variables shape the synaptic and membrane context in which a K^+^-dependent voltage change acts. One mechanistic implication of these separate findings is that K^+^-dependent depolarization may interact with voltage-dependent Mg^2+^ block, Ca^2+^-dependent transmitter release, and pH-dependent receptor and axonal excitability. How these interactions unfold during ordinary sustained cognitive demand has not been established within the literature examined for this Review. Extracellular K^+^ should therefore not be interpreted in isolation.

### 4.2. Astrocyte Membrane Potential and the Transmembrane Na^+^ Gradient Shape K^+^ and Glutamate Transport

Glutamate is not included as a core variable in the operational definition of the interstitial ionic state, but its extracellular dynamics are closely coupled to K^+^. Kir4.1-dependent K^+^ permeability helps maintain a hyperpolarized astrocyte membrane potential. This membrane potential, together with the transmembrane Na^+^ gradient, provides the electrochemical driving force for electrogenic glutamate uptake through excitatory amino acid transporters.

Fine astrocytic processes do not remain at a fixed membrane potential during neural activity. Genetically encoded voltage indicators revealed pathway-specific depolarization of peripheral astrocytic processes during local neural activity. This depolarization slowed glutamate clearance and enhanced neuronal glutamate responses [35]. Both perisynaptic K^+^ elevation and electrogenic glutamate transport contributed to the voltage change.

Receptor-initiated intracellular signaling can exert a different effect. In hippocampal slices, activation of the group I metabotropic glutamate receptor–protein kinase C pathway increased astrocytic glutamate-transporter currents and inward K^+^ currents [13]. These findings describe processes operating under different conditions: activity-dependent membrane depolarization can immediately reduce the driving force for glutamate uptake, whereas receptor-initiated signaling can increase transporter-associated and K^+^ currents.

Actual K^+^ and glutamate transport therefore depend not only on channel or transporter abundance, but also on astrocyte membrane potential, the transmembrane Na^+^ gradient, receptor signaling, and the energy required to restore ionic gradients. Recent multiphoton fluorescence-lifetime imaging revealed marked intercellular heterogeneity in astrocytic Na^+^ concentrations in mouse forebrain slices, with a broad distribution also observed in anesthetized cortex in vivo, and substantial subcellular heterogeneity within individual astrocytes in acute slices. In acute hippocampal slices from P14–20 BALB/c mice of both sexes, perfusion with 10 mM K^+^ for 2 min decreased astrocytic Na^+^ in somata and processes; the magnitude of the decrease was strongly correlated with baseline Na^+^, consistent with heterogeneity in K^+^-induced Na^+^/K^+^-ATPase activation [36]. Because this high-K^+^ manipulation was performed in juvenile acute slices and exceeded physiological wake-associated changes, it provides mechanistic evidence for heterogeneity in ion handling rather than a direct model of cognitive fatigability.

Astrocytic Ca^2+^ or cAMP signaling is therefore not equivalent to preserved K^+^ or glutamate transport. A working hypothesis is that, if sustained cognitive demand alters astrocyte membrane potential or intracellular Na^+^, a Ca^2+^ or cAMP signal of similar magnitude could produce a different transport response from that observed before the demand. Within the literature examined for this Review, no direct test of this proposed connection during ordinary sustained cognitive demand was identified. Astrocyte function should therefore be assessed from membrane potential, intracellular Na^+^, and actual K^+^ and glutamate transport rather than from a single Ca^2+^ or cAMP measure.

### 4.3. Extracellular Space Volume Shapes Changes in K^+^ Concentration

Extracellular K^+^ concentration depends on both the amount of K^+^ entering the extracellular compartment and the volume over which that K^+^ is distributed. The same K^+^ flux produces a larger concentration change in a smaller extracellular space and a smaller change in a larger space. Extracellular space volume is therefore a determinant of how ionic movement is converted into concentration.

Wake-associated shrinkage of extracellular space occurs together with increased extracellular K^+^ and pH and decreased extracellular Ca^2+^ and Mg^2+^ [7]. The higher extracellular K^+^ concentration of wakefulness need not therefore reflect ionic flux alone; a smaller distribution volume may also contribute.

Raising extracellular K^+^ in hippocampal CA1 produced preferential volume increases in astrocytes rather than neurons. The initial astrocyte swelling persisted in aquaporin-4-deficient tissue and was not clearly reduced by 100 μM BaCl_2_-mediated inhibition of inwardly rectifying K^+^ channels, whereas Na^+^/K^+^-ATPase inhibition attenuated it [16]. In this preparation, K^+^-evoked astrocyte swelling was linked to ion uptake and the resulting osmotic gradients. These findings do not establish a necessary role for aquaporin-4 or an isolated Kir4.1 mechanism in the initial swelling response. Subsequent work in acute hippocampal slices found that AQP4 deletion attenuated astrocytic swelling during exposure to 10 mM extracellular K^+^ but did not reduce peak swelling during exposure to 50 mM K^+^, indicating that AQP4 involvement depends on the magnitude and protocol of the K^+^ challenge [37].

In aquaporin-4-deficient mice, high-frequency synaptic stimulation altered extracellular space dynamics and produced a larger peak extracellular K^+^ concentration. The post-stimulus rate of K^+^ recovery, however, was not clearly different [17,18].

Water movement coupled to ion transport, astrocyte volume changes, extracellular space shrinkage, aquaporin-4-mediated water permeability, and K^+^ movement are related but nonidentical processes. These findings were obtained in brain slices using high-frequency stimulation and K^+^ conditions above those of ordinary wakefulness and cannot be transferred directly to sustained cognitive demand. They nevertheless show why a resting K^+^ concentration alone cannot establish restoration of the broader extracellular milieu. If extracellular space remains reduced after demand, the same ionic flux during renewed activity could produce a larger local concentration transient. Whether this occurs after ordinary sustained cognitive demand remains untested.

### 4.4. Sustained Ion Regulation Requires Metabolic and Vascular Support

Regulation of K^+^ and glutamate involves more than passive movement through channels such as Kir4.1. It requires Na^+^/K^+^-ATPase activity, Na^+^-dependent glutamate transport, and restoration of ionic gradients, all of which depend on a continuing supply of metabolic substrates.

As cortical activity increases with arousal, intracellular lactate in astrocytes decreases while extracellular lactate increases. This response is attenuated by β-adrenergic receptor blockade or depletion of brain glycogen [14]. Extracellular adenosine also promotes glucose metabolism and lactate release through astrocytic A_2_B receptors and the cAMP–protein kinase A pathway. Astrocyte-specific loss of A_2_B receptors impaired synaptic function, plasticity, memory, and sleep, particularly when energy demand was high or substrate supply was restricted [15].

These studies do not establish astrocyte-derived lactate as the brain’s sole energy source. They support the more limited conclusion that astrocytic metabolic pathways can become particularly relevant when energy demand is high or substrate supply is restricted, and that ATP-dependent pumps and transporters must be matched by adequate substrate delivery.

The present hypothesis does not require global depletion of brain adenosine triphosphate. Sustained cognitive demand could increase local or regional requirements for Na^+^/K^+^-ATPase activity, restoration of ionic gradients, glycogen use, and glucose and lactate metabolism while whole-brain energy remains available. Within the literature examined for this Review, a progressive increase in these requirements has not been demonstrated directly during ordinary sustained cognitive demand.

Metabolic support also depends on blood flow that supplies oxygen and substrates. The initial vasodilation evoked by brief sensory stimulation was preserved when IP_3_R2-dependent astrocytic Ca^2+^ responses were reduced [38]. In a separate study, suppression of astrocytic Ca^2+^ signaling during sustained stimulation selectively reduced the later component of the blood-flow response [39]. Endothelial mechanisms, including caveolae in central nervous system arterioles, also make substantial contributions to neurovascular coupling [40]. Blood-flow changes alone therefore cannot identify the functional state of astrocytes or the adequacy of K^+^ regulation.

Ion transport, metabolic support, vascular delivery, and water movement are coupled but not interchangeable processes, and they may evolve on different time scales. The unresolved question is whether their post-demand trajectories converge into an extracellular and cellular state capable of supporting rest, sleep, or renewed cognitive demand.

## 5. Recovery After Sustained Cognitive Demand: Reconfiguration of the Extracellular Milieu and Cellular Responsiveness

After sustained cognitive demand ends, neuromodulatory activity, astrocytic intracellular signaling, ion transport, energy metabolism, and water movement need not converge on the next functional state at the same rate. Each process follows its own trajectory and may retain effects of recent neural activity.

Recovery therefore cannot be inferred from a single variable, such as extracellular K^+^, returning to its pre-demand value. Two questions must be distinguished: whether the extracellular milieu has been reconfigured into a state suitable for rest, sleep, or renewed cognitive demand, and whether neurons and astrocytes have recovered the responsiveness required to operate within that milieu.

### 5.1. Recovery Is Not a Simple Reversal of Demand-Induced Changes

Human studies reveal at least two distinct forms of recovery-related dynamics. In a rest–task–rest design using working memory, endogenous brain activity required several minutes after task completion to approach its pre-task temporal organization, and this recovery was slower after the higher-load condition [3]. This finding directly shows that the neurophysiological effects of cognitive demand can persist into the post-task period.

A separate electroencephalography study examined performance during a continuous 120 min cognitive task. Performance followed a nonlinear trajectory, including a partial rebound after an initial decline while the task was still in progress. This within-demand behavioral rebound was associated with increased temporal variability in alpha-band functional connectivity in the frontoparietal network; the interaction between this variability and alpha power predicted the behavioral rebound [41]. The association and prediction are empirical findings, whereas the interpretation that they reflect active network reconfiguration is mechanistic.

These studies address different phases of the temporal process. The first demonstrates post-task persistence of altered brain dynamics, whereas the second demonstrates a partial behavioral rebound during continuing demand. Neither study measured the extracellular milieu or astrocyte function. Together, they show that behavioral improvement and neurophysiological change need not follow a simple mirror-image reversal of the preceding demand.

In this Review, physiological recovery refers to reconfiguration of the extracellular milieu, neuromodulatory input, and neuronal and astrocytic states into a configuration capable of supporting subsequent activity. Functional recovery refers to the return of accuracy, reaction time, response-time variability, or related behavioral measures during renewed demand to a prespecified range.

Within physiological recovery, this Review distinguishes reconfiguration of the extracellular milieu from recovery of cellular responsiveness to a standardized input under comparable extracellular conditions. Subjective recovery refers to a reduction in the experienced feeling of fatigue and should be distinguished from both physiological and functional recovery.

Physiological and functional recovery can dissociate. Functional recovery does not by itself establish physiological recovery, because behavioral improvement may be supported by compensatory neural recruitment or metabolic support while the underlying physiological state remains altered. Conversely, physiological recovery need not require every variable to return to a single pre-demand value, because rest, sleep, and renewed cognitive demand may require different extracellular and cellular configurations. Recovery is therefore better characterized as a trajectory toward a state capable of supporting the next activity than as a return to a universal baseline.

### 5.2. Temporal Features of Neuromodulatory Input and Astrocytic Responses

One potential source of post-demand history dependence is the differential temporal integration of neuromodulatory input by astrocytic second messengers. Astrocytic Ca^2+^ and cAMP responses can track different temporal features of noradrenergic activity.

In awake mice, an abrupt facial air puff produced transient noradrenaline release and a rapid, large astrocytic Ca^2+^ increase without a detectable cAMP response. Repeated aversive stimulation associated with prolonged vigilance instead produced a gradual and longer-lasting cAMP increase [42]. Optogenetic stimulation of noradrenergic axons likewise showed that shorter inputs were sufficient to elicit astrocytic Ca^2+^ responses, whereas cAMP responses required longer or more sustained input.

Ca^2+^ and cAMP therefore provide temporally distinct rather than redundant readouts of noradrenergic input. Under the conditions examined, Ca^2+^ responded to briefer input, whereas cAMP integrated more sustained input. Prolonged artificial activation of astrocytic stimulatory G-protein signaling also reduced glycogen content [42]. This manipulation links sustained intracellular signaling to use of an energy reserve, but it does not reproduce ordinary sustained cognitive demand.

These findings do not justify classifying Ca^2+^ as beneficial and cAMP as harmful, or vice versa. They suggest that the timing and persistence of intracellular responses should be interpreted in relation to the neuromodulatory input that elicited them. The working hypothesis is that, if this temporal correspondence remains altered after demand, reconfiguration of the extracellular milieu could be delayed. Within the literature examined for this Review, no direct test of this sequence during ordinary cognitive fatigability was identified.

Post-demand Ca^2+^ or cAMP persistence would therefore be a temporal signature rather than a direct measure of recovery or dysfunction. Its functional significance would need to be evaluated in relation to astrocyte membrane potential, intracellular Na^+^, K^+^ and glutamate transport; and metabolic state.

### 5.3. Recent Activity History Alters Cellular Responsiveness

Astrocytic responses do not begin from an identical cellular state on every occasion. In hippocampal CA1 of awake mice, population astrocytic Ca^2+^ activity reflected neuronal activity, movement, and pupil changes over the preceding several seconds [43]. Within comparable low-arousal events, higher astrocytic Ca^2+^ activity during the preceding period was associated with weaker centripetal propagation of the subsequent Ca^2+^ event.

These findings establish that astrocytic Ca^2+^ dynamics depend on recent cellular and behavioral history as well as on immediate input. The demonstrated history dependence operates over seconds, however, and does not directly establish corresponding changes in K^+^ or glutamate transport over the tens of minutes to hours relevant to cognitive fatigability.

In Drosophila, adrenergic G-protein-coupled receptor signaling in astrocytes gated subsequent responses to glutamate, acetylcholine, and γ-aminobutyric acid [44]. Related effects were observed in cultured mammalian astrocytes, but whether the same mechanism operates in the intact mammalian brain remains unresolved.

These studies motivate a distinction between the extracellular milieu and cellular responsiveness. Cellular responsiveness refers here to the membrane-potential change, firing, and synaptic transmission evoked by a standardized input in neurons, and to K^+^ and glutamate transport evoked by a standardized input in astrocytes. It is distinct from cognitive performance.

A working hypothesis is that residual changes in neuronal Na^+^ channel availability, intracellular Cl^−^ homeostasis, or Na^+^/K^+^-ATPase activity, or in astrocyte membrane potential, intracellular Na^+^, transport function, or metabolic state, could alter cellular responses even after the extracellular milieu has approached a suitable reference configuration. The inferential distance remains substantial: the present evidence concerns second-scale Ca^2+^ history dependence and, in one case, principally Drosophila. Whether comparable history dependence persists over minutes to hours in the intact mammalian brain remains untested. The remaining gap is therefore not whether recent history matters, but which processes can retain it over minutes to hours.

### 5.4. Sleep and Candidate Temporal Integrators

Sleep and wakefulness are accompanied by distinct extracellular milieus. The transition between them involves coordinated changes in extracellular K^+^, Ca^2+^, Mg^2+^, pH, and extracellular space volume, and experimentally imposing a wake-like ionic composition during sleep shifts local cortical activity toward a wake-like pattern [7]. Sleep therefore provides a physiological context in which the extracellular milieu transitions between distinct brain states.

Astrocytic Ca^2+^ activity does not cease during sleep. In freely behaving mice, astroglial Ca^2+^ activity was higher during wakefulness and lower during sleep, while activity during non-rapid-eye-movement sleep varied with sleep need. Conditional reduction in stromal interaction molecule 1 in astrocytes impaired the homeostatic sleep response after sleep deprivation [45]. These findings support a contribution of astroglial Ca^2+^ signaling to sleep regulation, but they do not show that astrocytic Ca^2+^ directly drives the sleep-associated ionic transition.

Within the present framework, sleep may provide an opportunity for the extracellular milieu, neuromodulatory systems, and metabolic processes established during wakefulness to be reconfigured for subsequent activity. Cognitive fatigability after sleep loss could therefore involve incomplete state transition, but this remains a working hypothesis.

Within the literature examined for this Review, no direct evidence was identified that sleep-associated changes in the interstitial ionic state determine cognitive fatigability on the following day. Establishing such a connection would require showing that sleep manipulations alter the post-demand trajectory of the extracellular milieu and that these changes are related to cognitive performance during renewed demand.

The timescale gap between second-scale ionic and intracellular signals and fatigability developing over tens of minutes to hours suggests a role for intermediate history-dependent processes. Here, candidate temporal integrator denotes a variable or process whose present state reflects recent activity over a longer interval and alters subsequent cellular or network responsiveness; it does not imply a single accumulating substance or an additional coordinate of the interstitial ionic state.

Neuronal intracellular Cl^−^ is one candidate. In mice, recent waking and local sensory use shifted the cortical reversal potential for γ-aminobutyric acid type A receptor-mediated currents in a depolarizing direction, and local manipulation of chloride cotransport altered low-frequency activity and modality-matched recognition after sleep deprivation [46]. A separate mouse study found a daily rhythm in cortical intracellular Cl^−^ and excitability that was not reducible to cumulative recent activity [47]. Sleep–wake-associated changes in intracellular Cl^−^ also altered subsequent membrane dynamics and the induction of synaptic plasticity in cortical preparations [48]. These studies position intracellular Cl^−^ as a history-dependent determinant of cellular responsiveness, not as evidence that extracellular Cl^−^ belongs in the core interstitial ionic state. Their paradigms involved sleep deprivation, circadian comparisons, anesthesia, or acute slices rather than ordinary sustained cognitive demand.

Extracellular adenosine may operate at a different level. In mice, basal-forebrain extracellular adenosine increased dynamically with neural activity; glutamatergic neurons contributed substantially to this signal, and their ablation reduced the increase and impaired sleep homeostasis [49]. This supports adenosine as an upstream or parallel sleep-homeostasis-related signal, not as a universal brain-wide accumulator or a direct mechanism of ordinary cognitive fatigability.

Local sleep provides a network-level candidate. In awake rats, local neuronal OFF periods increased with time awake and occurred more frequently before failed reaches [50]. Such events may contribute proximally to performance lapses, but they do not identify the ionic or cellular process that generated them, and sleep deprivation is not equivalent to sustained cognitive demand.

Sleep-dependent synaptic reorganization provides another parallel account. In mice, sleep promoted Homer1a-associated AMPA-receptor removal and dephosphorylation, with noradrenaline and adenosine exerting opposing influences on this process [51]. This establishes a sleep-related mechanism capable of altering subsequent synaptic responsiveness over hours, but it does not measure extracellular ions or directly explain cognitive fatigability.

These candidates need not be mutually exclusive with extracellular reconfiguration. A discriminating experiment could track extracellular K^+^ and pH, extracellular space volume, neuronal intracellular Cl^−^ or the reversal potential of γ-aminobutyric acid type A receptor-mediated currents, local field potentials and unit activity, and renewed-demand performance across the same or matched demand–recovery protocol. Support for the extracellular component would require its trajectories to add predictive value after the cellular and network variables are included; reciprocal analyses would test whether Cl^−^ or local OFF periods explain variance not captured by the extracellular model. Selective recovery-phase manipulations would provide a stronger causal test. No single experiment need measure every candidate, but the primary account and at least one discriminating measure should be prespecified.

### 5.5. Boundary Cases and Inflammatory Modifiers

The magnitude of astrocytic Ca^2+^ activity is not, by itself, a measure of astrocyte function or recovery. In mice overexpressing the astrocytic P2Y_1_ receptor, Ca^2+^ activity was reorganized into broader and longer-lasting waves rather than simply increased uniformly [52]. This study illustrates that the spatial and temporal organization of an intracellular signal may be more informative than event count or mean amplitude. It did not directly measure K^+^ or glutamate transport.

Severe neurophysiological perturbations provide boundary cases in which intracellular signaling, extracellular K^+^ clearance, and neuronal activity recovery can be compared. After photothrombotic ischemia, pan-adrenergic receptor antagonism facilitated normalization of extracellular K^+^ and recovery of neuronal activity [53]. Following KCl-induced cortical spreading depolarization, IP_3_R2 deficiency delayed extracellular K^+^ clearance and recovery of neuronal activity, whereas pan-adrenergic receptor antagonism facilitated recovery in both wild-type and IP_3_R2-deficient mice [54].

These results indicate that IP_3_R2-dependent signaling can contribute to recovery under cortical spreading depolarization, but the benefit of adrenergic receptor antagonism cannot be reduced to suppression of that signaling pathway. The pharmacological manipulation was not astrocyte-specific. Ischemia and cortical spreading depolarization are not direct models of ordinary cognitive fatigability; they are boundary cases showing that astrocytic Ca^2+^ magnitude alone does not predict extracellular K^+^ clearance or neuronal activity recovery.

Inflammatory signaling is likewise context dependent. In cultured cells and organotypic slices subjected to chronic activity blockade, astrocyte-derived tumor necrosis factor was required for homeostatic synaptic plasticity that compensated for reduced activity [55]. Under autoimmune inflammatory conditions, by contrast, tumor necrosis factor reduced glutamate-aspartate transporter expression and glutamate uptake [56]. Interleukin-1β reduced Kir4.1 expression in cultured and disease-related preparations [57].

These effects were observed mainly over hours to days and under culture, autoimmune, or other pathological conditions. Within the literature examined for this Review, no direct evidence was identified that neuroinflammation explains transient cognitive fatigability produced by tens of minutes of ordinary cognitive demand. In this framework, inflammatory signaling is therefore treated as a modifier that may alter extracellular regulation and recovery rather than as the established central mechanism of acute cognitive fatigability.

### 5.6. Two Routes to Delayed Recovery

As schematized in Figure 1, delayed recovery after sustained cognitive demand may arise through two non-exclusive routes: incomplete reconfiguration of the extracellular milieu and incomplete recovery of neuronal and astrocytic responsiveness.

First, the extracellular milieu may remain incompletely reconfigured for the state that follows the demand. Depending on the recovery interval, the relevant target may be a state suitable for rest, sleep, or renewed cognitive demand. If renewed demand is imposed before this transition is complete, information processing may begin in a milieu not yet suited to stable renewed performance (Figure 1A). Evaluation of this route should consider the trajectories of extracellular K^+^, pH, and extracellular space volume and, when relevant, Ca^2+^ and Mg^2+^, including the order and rate of their changes.

Second, the extracellular milieu may have approached a prespecified reference configuration while residual cellular changes persist. Incomplete recovery of neuronal Na^+^ channel availability, intracellular Cl^−^ homeostasis, Na^+^/K^+^-ATPase activity, astrocyte membrane potential, intracellular Na^+^, Kir4.1- and EAAT-mediated transport, or metabolic state could prevent a standardized input under comparable extracellular conditions from evoking the corresponding reference response in membrane potential, spiking, synaptic transmission, or K^+^ and glutamate transport (Figure 1B).

Because these routes may coexist, failure to detect a difference in the measured extracellular variables should not be used post hoc to shift the explanation to altered cellular responsiveness. The two routes and the measurements used to define them should be prespecified and evaluated independently.

Recovery assessment should therefore combine the multivariable post-demand trajectory of the extracellular milieu with a standardized-input test of neuronal and astrocytic responsiveness. These physiological measures can then be related to cognitive performance during renewed demand after a defined recovery interval.

## 6. An Integrated Model and Testable Predictions

A hypothesis linking cognitive fatigability to the extracellular milieu cannot be evaluated by simply juxtaposing relationships observed in different experimental preparations. It must be formulated as a temporal model spanning sustained cognitive demand, post-demand recovery, and renewed demand, with prespecified outcomes that can support, narrow, or weaken it.

An increase in extracellular K^+^ during cognitive demand would not, by itself, support the hypothesis, because physiological K^+^ elevation also accompanies arousal and movement. Similarly, increased astrocytic Ca^2+^ after demand cannot by itself distinguish adaptive signaling, incomplete recovery, and dysfunction. The decisive question is whether and how sustained cognitive demand alters the capacity to maintain and reconfigure the extracellular milieu, and whether any such alteration predicts physiological recovery and cognitive performance during renewed demand.

Table 1 organizes the evidence by inferential distance and does not imply that the integrated hypothesis has already been tested. It summarizes the evidence hierarchy informing the working hypothesis and the principal limitation at each level.

### 6.1. A Temporal Model from Cognitive Demand Through Recovery and Renewed Demand

Figure 2 summarizes a proposed temporal model linking sustained cognitive demand to post-demand recovery and renewed demand. The model is a working hypothesis assembled from findings obtained across distinct experimental paradigms. The arrows represent proposed temporal relationships and do not imply that each transition has been demonstrated within a single study.

In Figure 2, the pre-demand reference state denotes the prespecified reference condition for the task and brain state under study, not a universal fixed extracellular state. At demand onset, the model proposes that neuromodulatory input and neuronal activity contribute to the formation of a demand-adapted extracellular milieu, including a physiological change in extracellular K^+^. A modest K^+^ elevation can depolarize neurons and enhance their responses to a given input. Ion and glutamate transport, together with the metabolic support required for these processes, may therefore help preserve information processing during the initial phase of demand.

As demand continues, the model further proposes that sustaining comparable information processing may require increasing engagement of K^+^ and glutamate transport, restoration of ionic gradients, and metabolic support. Cognitive performance may remain apparently stable while the demands placed on these regulatory processes increase.

If these demands approach the momentary capacity of the relevant regulatory systems, neuronal responses may become more variable in magnitude and timing. At the behavioral level, the model predicts that cognitive fatigability will be expressed not as a uniform reduction in neural activity, but as increasing reaction-time variability, transient attentional lapses, and fluctuations in accuracy.

After the demand ends, neuromodulatory input, extracellular K^+^, astrocyte membrane potential, intracellular Na^+^, ion transport, energy metabolism, and water movement need not follow the same recovery trajectory. As defined in Section 5.6, post-demand recovery may be limited by incomplete reconfiguration of the extracellular milieu, incomplete recovery of cellular responsiveness, or both. Renewed demand imposed before the extracellular milieu and cellular responsiveness have reached states adequate for the next task is predicted to produce earlier or greater fatigability.

The proposed sequence therefore comprises a prespecified pre-demand reference state, formation of a demand-adapted extracellular milieu, maintenance through ion transport and metabolic support, increasing instability of information processing, post-demand recovery, and renewed demand. Its defining feature is not accumulation of a single substance, but a history-dependent reduction in the capacity to maintain and reconfigure the extracellular milieu across successive brain states.

### 6.2. Three Predictions That Can Adjudicate the Working Hypothesis

#### 6.2.1. Recovery Trajectories Should Predict Renewed-Demand Performance Better than an Immediate Post-Demand Measurement

Two individuals, or two sessions within the same individual, may show the same extracellular K^+^ concentration and similar cognitive performance immediately after demand yet follow different recovery trajectories. Extracellular K^+^, pH, extracellular space volume, and other relevant variables may change at different rates, in different orders, or with different residual deviations from their prespecified reference configurations.

If sustained cognitive demand delays reconfiguration of the extracellular milieu, prespecified features of these trajectories should predict reaction time, trial-to-trial variability, attentional lapses, and accuracy during renewed demand more accurately than a single immediate post-demand K^+^ measurement. This predictive value should extend beyond pre-demand performance, the immediate post-demand behavioral state, and prespecified covariates such as arousal, movement, and sleep pressure. Where feasible, the comparison should be evaluated prospectively or in held-out data.

The recovery-centered component of the hypothesis would be weakened if prespecified recovery trajectories provided no additional out-of-sample predictive value after accounting for pre-demand performance, relevant covariates, and model complexity.

#### 6.2.2. Hypothesis-Relevant Extracellular Variables Should Add Predictive Value Beyond K^+^ Alone

Extracellular K^+^ is the primary variable for an initial test of the hypothesis because it acts directly on neuronal and astrocytic membrane potential, but it is not assumed to be sufficient. Its effects are conditioned by extracellular pH, extracellular space volume, and, when relevant to the experimental question, Ca^2+^ and Mg^2+^.

A multivariable extracellular model should therefore predict recovery and renewed-demand performance better than a K^+^-only model if these additional variables carry hypothesis-relevant information. The variables and trajectory features to be included should be specified before outcome analysis. Not every variable must be measured in every experiment; the choice should follow the mechanism being tested rather than an attempt to maximize model fit.

Models should be evaluated in independent data or by cross-validation and penalized for added complexity. Here, “outperform” means a prespecified improvement in predictive performance in held-out or prospectively collected data after accounting for model complexity. Suitable metrics include lower root-mean-square error or mean absolute error, or higher out-of-sample R^2^, for continuous outcomes and lower log loss or Brier score for binary lapse outcomes; improved in-sample fit alone is insufficient. If prespecified extracellular variables do not improve out-of-sample prediction beyond K^+^ alone, the multivariable claim should be narrowed. Such a result should not be rescued by post hoc substitution of unmeasured variables.

#### 6.2.3. Manipulating Ion Regulation or Cellular Responsiveness During Recovery Should Alter Subsequent Fatigability

The strongest causal test is a recovery-phase intervention that changes a prespecified physiological process after the initial demand while minimizing changes in neural activity during the demand itself.

To test delayed reconfiguration of the extracellular milieu, a recovery-phase intervention could accelerate or delay a prespecified component of K^+^ regulation, extracellular pH regulation, or extracellular space dynamics while non-target extracellular changes are measured concurrently. A causal contribution would be supported if verified acceleration or delay of milieu reconfiguration shifted renewed-demand fatigability in the corresponding predicted direction.

To test cellular responsiveness, a prespecified cellular process, such as neuronal Na^+^ channel availability, intracellular Cl^−^ homeostasis, Na^+^/K^+^-ATPase activity, astrocyte membrane potential, or Kir4.1- or EAAT-mediated transport, could be manipulated partially and within a restricted recovery window. Neuronal membrane potential, spike timing, synaptic transmission, and astrocytic K^+^ and glutamate transport should then be assessed using a standardized input while the relevant extracellular conditions are measured and matched as closely as possible.

Causal support requires verified target engagement together with a change in cognitive fatigability during renewed demand in the predicted direction. Where experimentally feasible, reversibility or rescue should strengthen the inference. A behavioral change without verified target engagement would not support the proposed mechanism. Conversely, if the targeted physiological process changes by a prespecified amount without altering renewed-demand performance, the corresponding causal component of the hypothesis would be weakened. The three predictions and the outcomes that would support or weaken each are summarized in Table 2.

### 6.3. Testing the Model Requires Within-Subject Tracking Across Demand, Recovery, and Renewed Demand

The experimental system should permit sustained cognitive demand, post-demand recovery, and renewed demand to be assessed within the same subject without tissue injury or extreme metabolic disruption. The rodent continuous performance test is one candidate: extended sessions produced time-on-task-related performance decrements in male mice that were not explained solely by physical disengagement, although the pattern was not equivalent across sexes and experimental conditions [58]. A core design should include a pre-demand reference period, an immediate post-demand measurement, repeated measurements during recovery, and a standardized renewed-demand phase.

Cognitive fatigability should be analyzed as a within-subject trajectory rather than as a single average or an assumed linear slope. Prespecified features may include an initial period of preserved performance, a change point, subsequent decline, trial-to-trial variability, misses, and false alarms. Potential confounds should also be specified before analysis. These include movement and task engagement, reward and feeding state, circadian phase and sleep pressure, and physiological variables such as respiration, body temperature, and blood flow. Sleep and quiet wakefulness during the recovery interval should be distinguished because they are associated with different extracellular milieus.

A staged mechanistic design is preferable to measuring every variable at once. An initial test could combine the time course of extracellular K^+^ with one neuronal readout, such as membrane potential or spiking, and cognitive performance. Astrocyte membrane potential, intracellular Na^+^, Ca^2+^, cAMP, glutamate, pH, extracellular space volume, or neuromodulatory input can then be added prospectively according to the mechanism under test.

Cellular responsiveness should be assessed with the same small standardized input before demand, immediately after demand, and during recovery, while the extracellular variables relevant to the hypothesis are measured concurrently. Causal manipulations should preferably be partial, restricted to adulthood, and limited in brain region and time. Target engagement and non-target changes in the extracellular milieu must be assessed. For example, stimulation of channelrhodopsin-2 expressed in astrocytes can itself raise extracellular K^+^ [59], so optical manipulations require concurrent monitoring of non-target ionic changes, cell volume, energetic load, and temperature.

Direct extracellular K^+^ and pH measurements with ion-selective microelectrodes provide local temporal resolution but are vulnerable during prolonged recordings to drift, reference instability, tissue disturbance, and point sampling [60]. Real-time iontophoresis with tetramethylammonium can estimate local extracellular-space volume fraction and tortuosity [7], but its application during behavior is constrained by the need for stable source–sensor geometry. Genetically encoded extracellular K^+^ indicators require in situ calibration and controls for pH sensitivity, motion, photobleaching, and long-term signal stability. The membrane-targeted indicator RGEPO1 has enabled extracellular K^+^ imaging in awake mice during seizure-related activity [61], but its validity for smaller physiological changes across prolonged cognitive-demand and recovery protocols remains to be established. The astrocytic voltage measurements cited here were obtained mainly in slices, whereas the cited in vivo astrocytic Na^+^ measurements were obtained under anesthesia [35,36]; both therefore require separate validation for prolonged awake cognitive sessions.

The direct ionic predictions of this Review are presently most feasible in rodent models. In humans, repeated behavioral measurements and electroencephalographic, magnetoencephalographic, or magnetic-resonance trajectories can test whether post-demand brain dynamics predict renewed-demand performance, but these are systems- or tissue-level counterparts rather than direct measurements of local cortical interstitial ionic composition. Scalp slow-wave activity likewise does not by itself establish local neuronal OFF periods.

### 6.4. Alternative Mechanisms and Limitations

The extracellular-milieu framework is not an exhaustive account of cognitive fatigability. Neuronal Na^+^ channel inactivation, Na^+^/K^+^-ATPase-dependent afterhyperpolarization, synaptic-vesicle depletion, receptor desensitization, inhibitory networks, neuromodulatory systems, reward valuation, energy supply, and blood flow may all contribute. These mechanisms may operate through the extracellular milieu, in parallel with it, or partly independently of it. The present hypothesis does not replace them; it proposes a multivariable extracellular framework in which K^+^ serves as an anchor variable that may couple or condition several of these processes.

A capacity-constraint account would be favored if time-on-task performance instability persisted despite preserved task engagement, as observed in the rodent continuous performance test [58], and tracked physiological recovery trajectories; by contrast, rapid normalization after changing reward or effort costs without corresponding physiological recovery would favor a cost-revaluation account.

Rasmussen and colleagues previously synthesized evidence that interstitial ions regulate state-dependent neural activity [60]. The present Review does not claim novelty for that physiological premise; it extends the framework to the temporal relation among objective cognitive fatigability during sustained demand, post-demand reconfiguration of the extracellular milieu, recovery of cellular responsiveness, and performance during renewed demand. Its additional commitments are the distinction between the two recovery routes and the prespecified predictive and causal outcomes that would support or weaken the model. The scope also differs from the clinical Brain Fatigue Syndrome framework proposed by Johansson and Rönnbäck [62], which addresses persistent symptoms in disease or injury contexts rather than objective task-evoked fatigability and neurophysiological recovery over minutes to hours.

The candidate temporal integrators discussed in Section 5.4 operate at different explanatory levels and are not subsumed into the interstitial ionic state. Intracellular Cl^−^ can alter neuronal responsiveness, adenosine can act as an upstream or parallel extracellular signal, local sleep is a network phenotype, and synaptic homeostasis can provide a parallel sleep-dependent reorganization mechanism. Their contributions can be distinguished by asking whether extracellular trajectories, cellular-state variables, or local OFF periods provide incremental prediction after the others are included and by testing selective recovery-phase manipulations.

A mismatch between subjective fatigue and extracellular K^+^ or its recovery trajectory would not, by itself, falsify the hypothesis. The direct targets of explanation are cognitive fatigability and delayed neurophysiological recovery. Subjective fatigue is a distinct experience that integrates arousal, motivation, reward value, interoception, and affect.

A universal K^+^ threshold should not be expected across brain regions. The effects of extracellular K^+^ vary with brain region, cortical layer, astrocytic territory, vascular density, extracellular space volume, and recent neural activity. K^+^ changes during cognitive demand may also covary with movement, arousal, respiration, blood flow, and temperature. A correlation between extracellular K^+^ and cognitive performance would therefore be insufficient to establish causality.

The framework must be narrowed or revised if prespecified recovery trajectories add no predictive value, if a prespecified multivariable extracellular model does not outperform a K^+^-only model, or if verified recovery-phase target engagement fails to alter renewed-demand performance. Conversely, an isolated K^+^ change, a behavioral effect without target engagement, or a post hoc shift from unmeasured extracellular variables to unmeasured cellular responsiveness would not support the hypothesis. Support requires convergence among recovery trajectories, verified physiological changes, and cognitive performance during renewed demand.

## 7. Conclusions

This Review advances the working hypothesis that cognitive fatigability and delayed neurophysiological recovery may arise, in part, when sustained cognitive demand constrains the capacity to maintain an extracellular milieu that supports information processing and to reconfigure that milieu afterward. Delayed recovery may reflect incomplete reconfiguration of the extracellular milieu, incomplete recovery of neuronal and astrocytic responsiveness after the milieu has approached a suitable reference configuration, or both.

Within the literature examined for this Review, no direct test of this integrated hypothesis under ordinary sustained cognitive demand was identified. Support would require convergent predictive and causal evidence: prespecified recovery trajectories should improve prediction of renewed-demand performance beyond single-point measurements, multivariable extracellular models should outperform K^+^-only models, and a recovery-phase intervention with verified physiological target engagement should alter subsequent cognitive fatigability in the predicted direction. Understanding the proposed extracellular contribution to brain fatigue therefore requires more than quantifying how much demand preceded recovery; it requires determining how rapidly and flexibly the extracellular milieu can be reconfigured and cellular responsiveness can recover to support the next brain state.

## Figures and Tables

**Figure 1:**
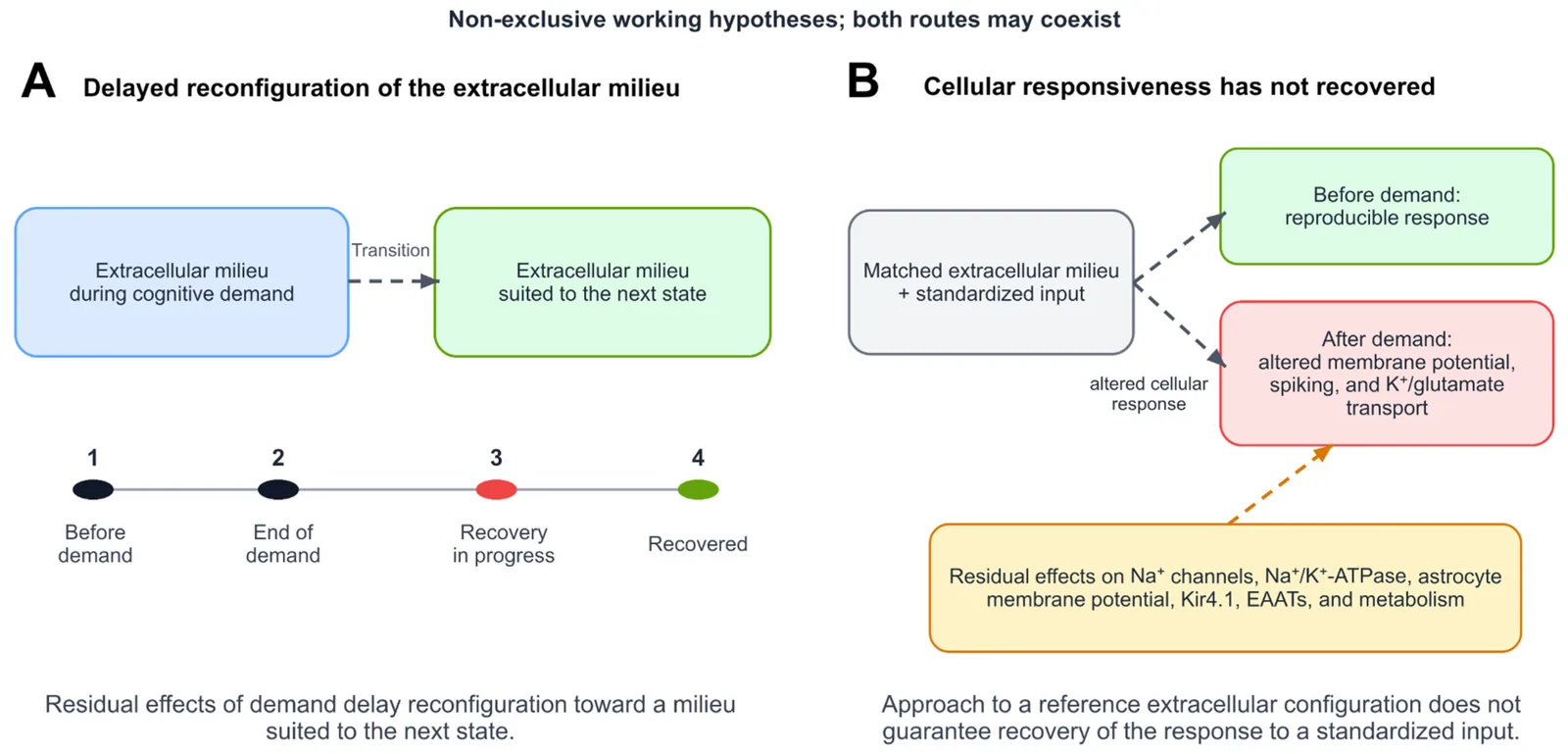
Conceptual diagram of two non-exclusive routes to delayed post-demand recovery. Left (**A**), the extracellular milieu may remain insufficiently reconfigured for the state that follows sustained cognitive demand. Right (**B**), the extracellular milieu may approach a prespecified reference configuration while residual changes in neurons or astrocytes prevent a standardized input from eliciting the corresponding reference response. “Extracellular milieu” refers to the multivariable extracellular conditions considered in this Review, including ionic composition and extracellular space volume, and not to K^+^ alone. The stages in Panel (**A**) are distinguished by numbers as well as by color. Both routes are working hypotheses. They may coexist, and the diagram does not imply a causal ordering between them.

**Figure 2:**
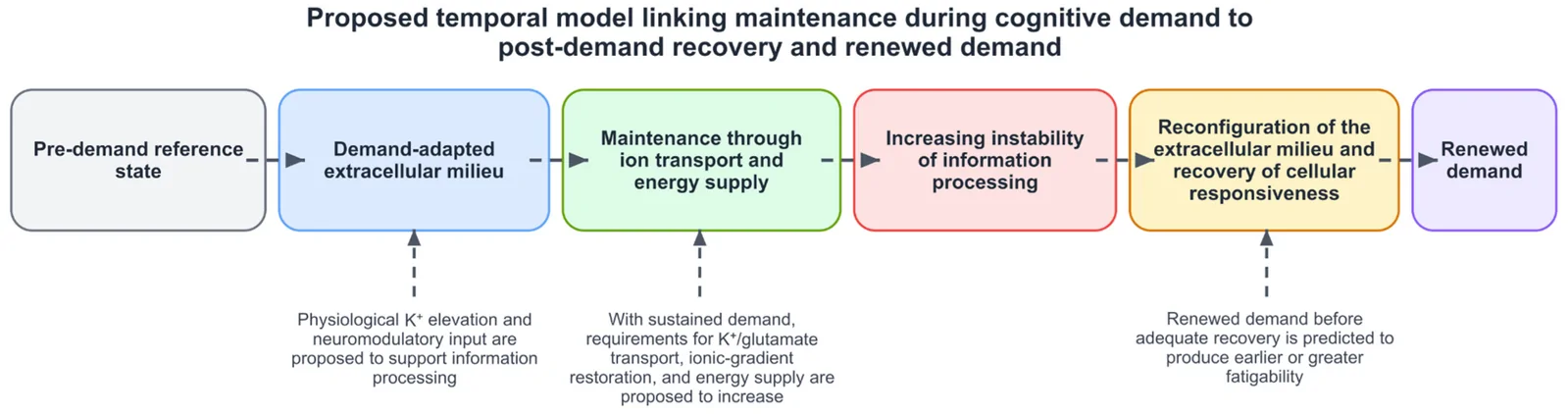
Proposed temporal model of cognitive demand, maintenance, recovery, and renewed demand. The pre-demand reference state denotes the prespecified reference condition for the task and brain state under study, not a universal fixed extracellular state. At demand onset, physiological K^+^ elevation and neuromodulatory input are proposed to contribute to a demand-adapted extracellular milieu that supports information processing. As demand continues, the engagement of K^+^ and glutamate transport, restoration of ionic gradients, and metabolic support is hypothesized to increase. If the demands placed on these processes approach their momentary regulatory capacity, information processing is predicted to become more variable. After demand, reconfiguration of the extracellular milieu and recovery of cellular responsiveness may proceed at different rates. Renewed demand before both are adequate for the next task is predicted to produce earlier or greater fatigability. Dashed arrows denote proposed temporal relationships; the arrows do not imply that each causal step has been demonstrated within a single experimental paradigm.

**Table 1:** Evidence Hierarchy Informing the Working Hypothesis and Its Principal Limitations.

Position in the Evidence Hierarchy	Representative Evidence or Proposed Relation	Role in This Review	Principal Limitations
Physiological principles	Coordinated changes in extracellular K^+^, Ca^2+^, Mg^2+^, pH, and extracellular space across sleep–wake states; state-linked K^+^ changes during movement; altered local neural activity after experimental manipulation of extracellular ionic composition. Representative references: [7,8,9].	Supports the premise that the extracellular milieu is dynamic and can shape the conditions for subsequent neural activity	Ordinary sustained cognitive demand and cognitive fatigability were not measured directly
Proximal cellular mechanisms	K^+^-dependent membrane-potential changes, firing-history-dependent Na^+^ channel availability, Na^+^/K^+^-ATPase-dependent afterhyperpolarization, and Kir4.1- and EAAT-dependent transport. Representative references: [8,22,23,24,25,26,27,28,29,30,31,32,33,34,35,36].	Provides cellular mechanisms through which the magnitude and timing of neural responses could become unstable	Evidence includes brain slices, high-frequency stimulation, developmental or genetic models, and relatively high K^+^ conditions
Mechanistic bridging evidence	Neuromodulatory regulation of astrocytic Ca^2+^ and cAMP, metabolism, water movement, extracellular space, and blood flow. Representative references: [10,11,12,13,14,15,16,17,18,35,36,37,38,39,40,42,43,44,45].	Provides candidate links between brain-state signals and maintenance or reconfiguration of the extracellular milieu	The full sequence has not been demonstrated within a single experimental paradigm
Boundary cases	Ischemia, cortical spreading depolarization, and inflammation-related changes in ion or glutamate transport. Representative references: [53,54,55,56,57].	Illustrates context dependence and dissociations among intracellular signaling, K^+^ clearance, and neuronal recovery	These conditions are not direct models of ordinary cognitive fatigability
Working hypothesis proposed here	Sustained cognitive demand may progressively constrain maintenance and post-demand reconfiguration of the extracellular milieu and delay recovery of cellular responsiveness	Integrates cognitive fatigability and delayed recovery into one testable temporal process	The integrated sequence has not been directly tested under ordinary sustained cognitive demand

**Table 2:** Core Predictions and Outcomes That Would Support or Weaken the Working Hypothesis.

Core Prediction	Measurements and Prespecified Analysis	Outcome Supporting the Hypothesis	Outcome That Would Weaken the Hypothesis
Recovery trajectories predict renewed-demand performance	Repeated within-subject measurements before demand, immediately after demand, during recovery, and during renewed demand. Prespecify recovery rate, order of change, time to a reference range, and residual deviation. Compare trajectory and immediate-measurement models in held-out data or participant- or session-blocked cross-validation.	Prespecified features of multivariable recovery trajectories predict renewed-demand performance in held-out or prospectively evaluated data beyond an immediate post-demand measurement, pre-demand performance, and relevant covariates	Recovery trajectories provide no additional out-of-sample predictive value after accounting for pre-demand performance, relevant covariates, and model complexity
A multivariable extracellular model outperforms a K^+^-only model	Compare K^+^-only and prespecified multivariable models using the same observations, covariates, and data partitions. Evaluate complexity-adjusted out-of-sample performance with an endpoint-appropriate metric, such as root-mean-square error, mean absolute error, out-of-sample R^2^, log loss, or Brier score.	A model incorporating prespecified extracellular variables outperforms a K^+^-only model in independent data or cross-validation after penalizing added complexity	Adding extracellular pH, extracellular space volume, Ca^2+^, Mg^2+^, or other prespecified variables does not improve out-of-sample prediction beyond K^+^ alone
Recovery-phase manipulation alters subsequent fatigability	Manipulate a prespecified recovery-phase process; verify target engagement; assess cellular responsiveness with a standardized input; monitor non-target ionic and behavioral effects; and measure renewed-demand performance.	A recovery-phase intervention produces verified target engagement and changes renewed-demand fatigability in the predicted direction, with reversibility or rescue where feasible	The targeted physiological process changes by a prespecified amount, but renewed-demand cognitive performance remains unchanged

## Data Availability

No new data were created or analyzed in this study. Data sharing is not applicable to this article.

## Abbreviations

The following abbreviations are used in this manuscript:

Abbreviation	Definition
AMPA	α-amino-3-hydroxy-5-methyl-4-isoxazolepropionic acid
ATP	adenosine triphosphate
cAMP	cyclic adenosine monophosphate
EAAT	excitatory amino acid transporter
IP_3_R2	inositol 1,4,5-trisphosphate receptor type 2
KCC2	K^+^–Cl^−^ cotransporter 2
Kir4.1	inwardly rectifying potassium channel 4.1
MRS	magnetic resonance spectroscopy
NMDA	N-methyl-D-aspartate
P2Y_1_	P2Y purinoceptor 1
